# Cost-effectiveness of interventions to improve case finding for tuberculosis: developing consensus to motivate investment

**DOI:** 10.1186/s44263-023-00024-3

**Published:** 2023-10-11

**Authors:** David W. Dowdy, Hojoon Sohn

**Affiliations:** 1 Department of Epidemiology, Johns Hopkins Bloomberg School of Public Health, Baltimore, MD USA; 2 Department of Preventive Medicine, Seoul National University College of Medicine, Seoul, Republic of Korea; 3 Department of Human Systems Medicine, Seoul National University College of Medicine, Seoul, Republic of Korea

**Keywords:** Tuberculosis, Active case finding, Cost-effectiveness, Consensus building

## Abstract

To better evaluate the cost-effectiveness of active case finding for tuberculosis, a framework for estimating long-term cost and impact is needed. We outline such a framework and highlight the need for consensus estimates of which costs to measure; averted morbidity, mortality, and transmission; measurable short-term outcomes; and meaningful cost-effectiveness thresholds.

## Background

It is increasingly recognized that global aspirations for ending tuberculosis (TB) cannot simply rely on improving health system infrastructure and introducing new technologies [1]. With more than 30% of TB cases unnotified every year [2], it is essential to also expand and innovate strategies to find people with prevalent TB, especially at earlier stages of the disease. Unfortunately, such efforts at active case finding (ACF) remain vastly underutilized. For example, household contact investigation (one form of ACF) is broadly recommended but inconsistently performed in most high-burden settings. As a result, the world has met less than 5% of the 2018–2022 target for TB preventive treatment among household contacts aged 5 years or older [2]. Efforts to scale up community-based ACF are even less well actualized.

Lack of available resources is arguably the most common barrier cited to the broader implementation of ACF [3]. Mathematical models have suggested that ACF can be highly cost-effective if the cost per case detected and treated is under $1700 (in 2022 US dollars) in countries like India [4]—a threshold easily met in most of the 29 TB REACH Wave 5 projects analyzed in 2022 [5]. However, economic evaluations of ACF are sparse, and while a return-on-investment analysis suggested that each dollar invested in a community health worker (CHW)-based ACF strategy in Vietnam could yield over $30 in return [6], a generalizable investment case for ACF has yet to be formulated. It therefore merits consideration as to why our thinking about the economics of TB ACF is behind that of other health interventions—and how this situation can be improved.

## Toward a consensus framework

We argue that a major barrier to effective economic evaluation of ACF is the lack of a consensus framework for estimating the long-term impact and thus also the incremental cost-effectiveness of ACF. Specifically, it is widely understood that people with TB who are detected earlier through ACF might be detected later through routine clinical care, and at much lower cost. As such, simply measuring the number of people diagnosed in an ACF program will overestimate the number of incremental diagnoses when compared to a standard of care with no ACF. However, the number of diagnoses is not the only relevant consideration. The timing of diagnosis is also important. Delayed diagnosis can result in an increased burden of post-tuberculosis sequelae, not to mention the risk of death if the diagnosis is too late to avert mortality. Furthermore, individuals with subclinical TB that could be detected through ACF likely contribute substantially to the transmission of *M. tuberculosis* [7]*.* It is therefore possible, if not likely, that early detection through ACF could avert an important burden of transmission, morbidity, and mortality, even in the absence of measurable impact on overall case detection or prevalence. Such early detection might also reduce costs, including catastrophic costs, to patients of TB treatment [8]. In other words, there has been no systematic attempt to quantify what would happen to people diagnosed with TB through ACF in terms of transmission, clinical outcomes, and patient-level costs, had ACF never been performed.

**Fig. 1:**
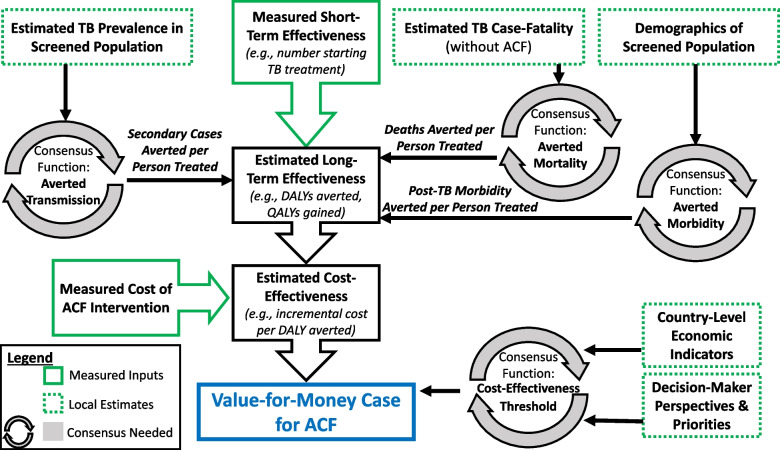
Components needed to make a strong value-for-money case to invest in active case finding (ACF) for tuberculosis. In general, ACF programs have capacity to evaluate their short-term outcomes (such as the number of diagnoses made or treatments initiated) and costs; these components are indicated by solid green boxes. Implementing partners can also generally estimate specific quantities regarding the populations being screened (shown in dotted green boxes). What is therefore needed is scientific consensus (shown in grey circular arrows) regarding which costs to measure and how to convert costs, short-term outcomes, and population characteristics into estimates of long-term effectiveness, cost-effectiveness, and judgments of whether cost-effectiveness estimates meet criteria for action. Abbreviations: DALY, disability-adjusted life year; QALY, quality-adjusted life year

In the absence of empirical data to directly answer this question, the development of scientific consensus would greatly benefit efforts to build an economic case for ACF (Fig. 1). This consensus should include the following components, each representing the current state of the scientific literature and ideally estimated for different emblematic types of ACF (e.g., household contact investigation, screening in congregate settings, community-based screening):

- Costs to be measured: intervention costs, inclusive of resources required for design, initiation, and maintenance at both site-specific and central levels;
- Short-term effectiveness outcome(s): outcome(s) that, if empirically measured, could be subsequently transformed/modeled into more generalizable estimates of cost-effectiveness;
- Averted transmission: estimated number of secondary cases likely to be averted, per short-term effectiveness outcome, as a function of underlying TB prevalence;
- Averted mortality: estimated risk of mortality among people with prevalent TB who are not detected by ACF, as a function of estimated TB case-fatality in the population;
- Averted morbidity: estimated lifetime burden of post-TB sequelae among people with prevalent TB who are not detected by ACF, as a function of population demographics and comorbidities (e.g., HIV);
- Cost-effectiveness/willingness to pay threshold: cost-effectiveness that would be sufficient to justify a strong investment case, according to underlying economic conditions and funder perspectives.

As depicted in Fig. 1, consensus on these elements would provide a pathway for implementers of ACF interventions to make a strong economic case for scale-up and/or sustainability. Specifically, implementers would be empowered to input their estimates of cost and short-term effectiveness (e.g., number of people started on TB treatment through ACF activities), as well as readily available estimates of local TB epidemiology (e.g., prevalence of TB in the screened population) and economic indicators (e.g., country-specific cost-effectiveness thresholds [9])—and rapidly obtain estimates of cost-effectiveness. These estimates could then be compared to consensus thresholds, ideally developed in consultation with relevant decision-makers, to evaluate whether ACF provides good value for money. Implementers and decision-makers would also be empowered to see how those estimates might change under different conditions—enabling, for example, benchmarks of post-implementation cost and effectiveness that must be met for funding to continue. If the functions underlying these consensus estimates were sufficiently simple, transparent, and easy to use, this process could be performed in a reproducible fashion and with relatively little reliance on outside technical expertise. Such consensus would also incentivize the standardized collection of agreed-upon short-term effectiveness measures and corresponding costs.

## Challenges

Despite these advantages of developing a consensus framework, it is reasonable to ask whether the current state of the science is sufficient to support such an effort. As described above, there is substantial uncertainty as to the long-term effects of early detection on transmission, morbidity, and mortality. This uncertainty could be formally incorporated into any decision-making framework. However, for many decision-makers, a consensus point estimate will still be highly relevant. If the current scientific consensus, based on the best available evidence, is that ACF is likely to have meaningful benefits and minimal harms for screened populations, it is important to develop the tools necessary to clearly convey that message.

In deciding whether to pursue such a consensus-building exercise, it is important to consider the anticipated messaging to funders and other decision-makers if a scientific consensus regarding the long-term effectiveness and thus cost-effectiveness of ACF is not pursued. Without such consensus, policy recommendations will remain weak and conflicting—with the near-certain result that global implementation of ACF will remain halfhearted and underfunded. The scientific community cannot reasonably criticize country-level policymakers and external donors for failing to prioritize ACF if we are not able to develop the consensus that would be needed to make a compelling economic case for implementing ACF in specific populations. If scientists, thought leaders, and advocates do not work together to develop the best available evidence-based consensus regarding the most likely long-term effects of ACF (recognizing that this consensus will change as new data emerge), then we are implicitly agreeing that ACF should not be an immediate priority for TB control.

## Conclusion: a call to action

ACF should not be held to an unreasonable standard of demonstrating population-level (i.e., epidemiological) impact to be worthy of investment. Trials evaluating the impact of ACF on population-level outcomes such as TB prevalence have been suggestive but not conclusive of epidemiological impact [10]. In evaluating cost-effectiveness and appropriateness for investment, however, it is reasonable to consider anticipated individual-level, rather than population-level, effectiveness. For example, strong investment/value-for-money cases have been made for TB diagnostic assays (e.g., Xpert MTB/RIF), treatment, and preventive treatment—none of which has been empirically demonstrated to reduce TB incidence or prevalence at the population level. Similarly, if the net anticipated benefits of ACF to those being screened are sufficient to justify the costs, broader implementation should be recommended, even if impacts on population-level epidemiology remain uncertain. And the likely benefits of early TB detection in terms of reduced mortality risk and fewer long-term sequelae are indeed substantial.

In summary, the failure to make a strong investment case for ACF has greatly hampered broader scale-up. Central to this investment case is an understanding of value-for-money (i.e., cost-effectiveness). Arguably the key missing piece in evaluating the cost-effectiveness of ACF is the lack of scientific consensus regarding likely long-term outcomes among people with TB who could be detected earlier through ACF but currently experience delayed detection through routine systems. Consensus-based, up-to-date estimates of costs to measure, averted morbidity, mortality, and transmission through ACF that can be coupled with measurable short-term effectiveness outcomes and meaningful cost-effectiveness thresholds would represent a major contribution to advocates and implementers trying to make a stronger case for investment. Without undertaking such an effort, we can expect continued rhetoric promoting the importance of ACF for global TB control, but without providing the tools needed to convince funders and decision-makers that ACF is a good use of extremely limited resources for health.

## Data Availability

Not applicable.
